# Nuclear retention of full-length *HTT* RNA is mediated by splicing factors MBNL1 and U2AF65

**DOI:** 10.1038/srep12521

**Published:** 2015-07-28

**Authors:** Xin Sun, Pan P. Li, Shanshan Zhu, Rachael Cohen, Leonard O. Marque, Christopher A. Ross, Stefan M. Pulst, Ho Yin Edwin Chan, Russell L. Margolis, Dobrila D. Rudnicki

**Affiliations:** 1 Department of Psychiatry and Behavioral Sciences, Division of Neurobiology, Johns Hopkins University School of Medicine, Baltimore, Maryland, USA; 2 Department of Neurology, Johns Hopkins University School of Medicine, Baltimore, Maryland, USA; 3 Department of Neuroscience, Johns Hopkins University School of Medicine, Baltimore, Maryland, USA; 4 Program of Cellular and Molecular Medicine, Johns Hopkins University School of Medicine, Baltimore, Maryland, USA; 5 Department of Neurology, University of Utah, Salt Lake City, Utah, USA; 6 Laboratory of Drosophila Research, School of Life Sciences, Faculty of Science, The Chinese University of Hong Kong, Shatin, N.T., Hong Kong SAR, China; 7 Guangdong-Hong Kong-Macau Institute of CNS Regeneration, Jinan University, Guangzhou, Guangdong, China

## Abstract

Huntington’s disease (HD) is caused by a CAG repeat expansion in the huntingtin (*HTT*) gene. Recent evidence suggests that HD is a consequence of multimodal, non-mutually exclusive mechanisms of pathogenesis that involve both HTT protein- and *HTT* RNA-triggered mechanisms. Here we provide further evidence for the role of expanded *HTT (expHTT)* RNA in HD by demonstrating that a fragment of *expHTT* is cytotoxic in the absence of any translation and that the extent of cytotoxicity is similar to the cytotoxicity of an expHTT protein fragment encoded by a transcript of similar length and with a similar repeat size. In addition, full-length (FL) *expHTT* is retained in the nucleus. Overexpression of the splicing factor muscleblind-like 1 (MBNL1) increases nuclear retention of *expHTT* and decreases the expression of expHTT protein in the cytosol. The splicing and nuclear export factor U2AF65 has the opposite effect, decreasing exp*HTT* nuclear retention and increasing expression of expHTT protein. This suggests that MBNL1 and U2AF65 play a role in nuclear export of *expHTT* RNA.

Huntington’s disease (HD) is caused by CAG repeat expansions in the huntingtin (*HTT*) gene^1^. While there is undoubtedly support for the role of expHTT protein in HD pathogenesis^2^, efforts to ameliorate HTT protein–induced toxicity have not yet yielded an effective treatment. Based on evidence from other CAG/CTG repeat expansion diseases, including spinocerebellar ataxias type 3 and 8 (SCA3 and SCA8, respectively)^3^,^4^,^5^ and the HD genocopy Huntington’s disease-like 2 (HDL2)^6^,^7^, multimodal pathogenesis, including RNA toxicity, is likely to be involved in HD^8^,^9^. Indeed, at least three mechanisms of *expHTT* RNA neurotoxicity have been proposed. First, RNA with sufficiently long stretches of CUG or CAG triplets forms hairpin structures^10^ leading to the sequestration of RNA-binding proteins (RBPs), including the splicing factor MBNL1^7^,^9^,^11^, with complications that include widespread splicing abnormalities. In support of this hypothesis, expanded *HTT (expHTT)* RNA in patient-derived HD fibroblasts aggregates into foci that co-localize with MBNL1, with the predicted consequence that at least some transcripts under MBNL1 control are misspliced^11^,^12^. Second, bidirectional *HTT* transcripts^13^ may provide a source of Dicer-generated CAG/CUG repeat siRNAs capable of targeting cellular transcripts containing complementary repeats, with potentially lethal consequences^8^,^14^. Third, repeat-associated non-ATG translation (RAN), an RNA-dependent mechanism triggered by structural abnormalities of repeat expansion-containing transcripts, may lead to expression of proteins containing expanded tracts in HD^15^. These three mechanisms are non-mutually exclusive and suggest that ignoring RNA-mediated pathogenic pathways in HD risks missing opportunities to develop novel therapies that will complement efforts aimed at reducing HTT protein-mediated neurotoxicity.

In the current study, we provide further evidence of the cytotoxicity of untranslated *expHTT* and describe how nuclear retention of *expHTT* is regulated, in opposite directions, by the splicing factors MBNL1 and U2 small nuclear ribonucleoprotein auxiliary factor 65 (U2AF65). We hypothesize that nuclear retention of *expHTT* is likely to lead to increased neurotoxicity, and therefore that pathways leading to this phenomenon may provide valuable therapeutic targets.

## Results

### An untranslatable *expHTT* RNA fragment is cytotoxic in the absence of both ATG- and non-ATG-initiated translation

**Figure 1:**
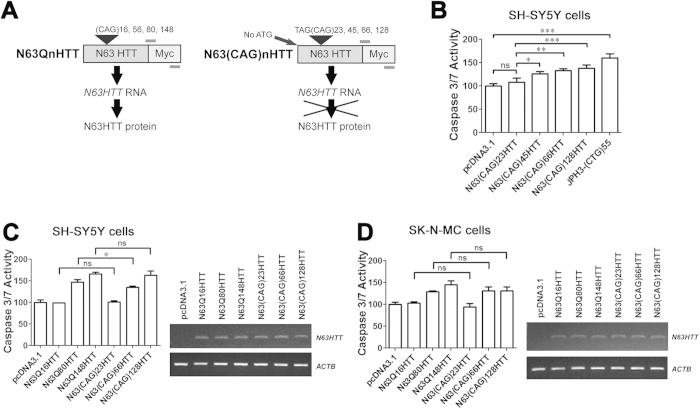
*ExpHTT* RNA contributes to HD neurotoxicity. (**A**) Schematic representation of N63QnHTT and N63(CAG)nHTT plasmids. (**B**) SH-SY5Y cells were transfected with N63(CAG)nHTT plasmids and, 72 hours post-transfection, cytotoxicity was assessed by caspase-3/7 activity assay. pcDNA3.1 plasmid and JPH3-(CTG)55 plasmid expressing non-translatable RNA with CUG repeat expansion were used as negative and positive controls, respectively. One-way ANOVA, n = 4 biological replicates. *P < 0.05, **P < 0.01, ***P < 0.001, ns = no significance. (**C**) SH-SY5Y cells and (**D**) SK-N-MC cells were transfected with N63QnHTT or N63(CAG)nHTT plasmids and, 72 hours post-transfection, cytotoxicity was assessed by caspase-3/7 activity assay. pcDNA3.1 plasmid was used as a negative control. Both experiments, one-way ANOVA, n = 4 biological replicates. *P < 0.05, ns = no significance. Comparable expression levels of exogenous *N63HTT* RNAs were confirmed by RT-PCR. Beta-actin (*ACTB*) transcript was used as a loading control.

An *HTT* exon 1 fragment encoding *expHTT* RNA with 80 CAG repeats is cytotoxic in SH-SY5Y cells^14^. To test the toxicity of untranslated *HTT* transcripts with CAG repeats ranging in length from 23 triplets (normal) to 128 triplets (extreme juvenile onset), and including a length of 45 triplets (typical adult-onset HD), we overexpressed truncated *HTT* RNA in the SH-SY5Y and SK-N-MC neuroblastoma cell lines. To obtain the non-translatable N63(CAG)nHTT construct, we modified a truncated N63QnHTT construct containing a sequence corresponding to the first 63 amino acids of HTT plus the repeat region^16^ by deleting the ATG 5′ to the repeat region and placing a STOP codon immediately 5′ to the repeat (Fig. 1A). Transfection of these untranslatable N63(CAG)nHTT constructs (with 23, 45, 66 or 128 CAG triplets), in a pcDNA3.1 vector, into SH-SY5Y cells resulted in repeat-dependent cytotoxicity, as measured by caspase-3/7 activity assay performed 72 hours after transfection (Fig. 1B). Recently, it was shown that, in addition to the toxic polyglutamine (polyGln), toxic polyalanine (polyAla) and polyserine (polySer) can be produced from *expHTT* by RAN translation in specific cell types^15^. To confirm that in our experiments cytotoxicity is indeed caused by toxic *HTT* RNA itself, we cloned untranslatable *N63(CAG)nHTT* sequences into a vector in which each sense-strand reading frame is tagged (N63(CAG)nHTT-RAN plasmid^15^) (Supplementary Fig. S1) and tested for the presence of RAN translation proteins in SH-SY5Y cells. With the exception of *N63(CAG)*_*150*_*HTT* RNA, which expresses a polyAla-containing RAN product, no evidence of RAN translation was observed in SH-SY5Y cells expressing *expHTT* (Supplementary Fig. S1). This confirms that *expHTT* RNA with a CAG repeat size within the range most frequently found in HD patients is cytotoxic in a neuroblastoma cell model.

### Untranslated *expHTT* RNA is a significant contributor to cytotoxicity in HD

We next tested the cytotoxicity of *expHTT* RNA, using expanded expHTT protein as a point of reference, and whether RAN translation contributes to *expHTT* RNA toxicity. We first determined that, unlike SH-SY5Y cells (Fig. S1B), SK-N-MC cells are permissive of RAN translation. Transfection of non-translatable N63(CAG)nHTT plasmids with 66 and 150 CAG triplets into SK-N-MC cells resulted in high levels of RAN translation products encoding polyAla. Expression of polyGln and polySer was observed following overexpression of 66 CAG triplets (Supplementary Fig. S1C). We therefore transfected SH-SY5Y cells with either non-translatable N63(CAG)nHTT plasmids (with 23, 66 or 128 CAG triplets) expressing only *HTT* RNA, or, as an approximate reference point for neurotoxicity, unmodified, translatable N63QnHTT plasmids (with 16, 80 or 148 CAG triplets) expressing both *HTT* RNA and protein. We used truncated HTT constructs for these experiments, as these constructs enabled us to control for levels of transcription and to measure cytotoxicity in an unbiased manner using chemical readouts. Although repeat lengths were not precisely matched between the constructs expressing translatable and non-translatable transcripts due to slight shifts in repeat lengths during plasmid preparation, similar levels of RNA were expressed from all HTT constructs and triggered comparable levels of cytotoxicity at each repeat length (Fig. 1C). We next performed the same experiment in SK-N-MC cells to determine if RAN translation significantly affects toxicity in this model. Once again, constructs expressing only *expHTT* RNA and constructs expressing both *expHTT* RNA and protein triggered similar levels of cytotoxicity (Fig. 1D). Taken together, the data indicate that *expHTT* RNA significantly contributes to cytotoxicity and that RAN translation does not add to cytotoxicity in a neuroblastoma model of HD. Whether this is true in the context of FL HTT and in other cell types remains to be examined.

### MBNL1 decreases foci formation of expanded full-length *HTT* RNA

**Figure 2:**
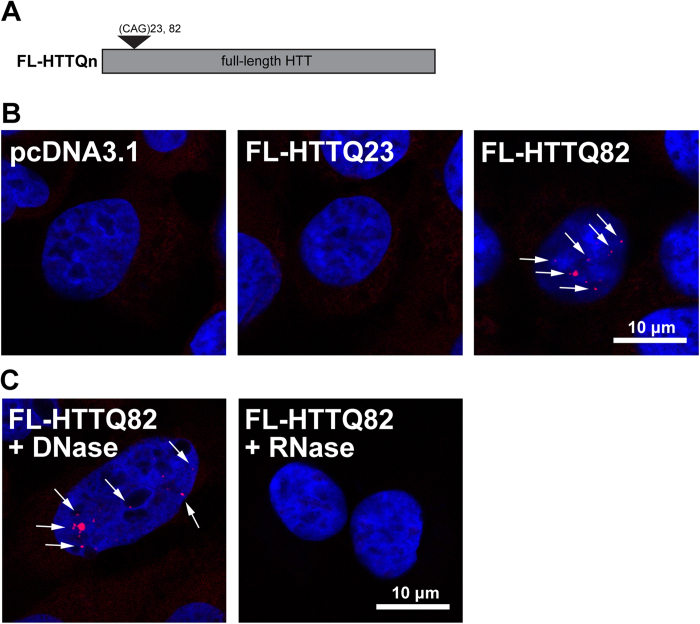
FL *expHTT* RNA forms nuclear foci in the SK-N-MC neuroblastoma cell line. (**A**) Schematic representation of FL-HTTQn plasmids. (**B**) Cells from the SK-N-MC neuroblastoma line were transfected with pcDNA3.1, FL-HTTQ23 or FL-HTTQ82 plasmids, and, 48 hours post-transfection, FISH was performed to detect CAG RNA foci using a 5′ Texas Red-labeled CUG20 riboprobe (red, arrows). Hoechst was used as a nuclear marker. Green fluorescence was imaged as a background control. FL *HTT* RNA with 23 CAG repeats (*FL-HTTQ23*) formed rare foci, whereas RNA foci were frequently formed by FL *HTT* RNA with 82 repeats (*FL-HTTQ82*). (**C**) SK-N-MC cells were transfected with FL-HTTQ82 plasmid, treated with DNase or RNase, and then subjected to FISH. Persistence of RNA foci after DNase treatment and absence of the foci following RNase treatment (arrows) confirms that the foci contain RNA. Scale bar, 10 μm.

Nuclear RNA foci, punctate aggregates of expanded RNA, are hallmarks of RNA-mediated toxicity^17^,^18^. In HD, RNA foci have been detected in HD patient–derived fibroblasts expressing 44, 68, 69 or 151 CAG triplets^11^,^12^. Using fluorescence *in situ* hybridization (FISH) with a 2′-*O*-methylated CUG riboprobe, we observed RNA foci in truncated N63HTT constructs expressed in SK-N-MC cells (Supplementary Fig. S2) and in the cortex of the N586-82Q transgenic mouse model of HD^19^ (Supplementary Fig. S2). Next we asked whether full-length (FL) *expHTT* RNA forms foci in neuronal cells, as it is possible that the additional sequence may influence the capacity of *expHTT* to form RNA foci. To test this, we overexpressed FL *expHTT* with 82 CAG triplets (FL-HTTQ82, Fig. 2A) in SK-N-MC cells. As predicted, FL *expHTT* RNA formed RNA foci (Fig. 2B) similar in appearance to those observed following expression of truncated *expHTT*, and resistant to DNase and sensitive to RNase treatment (Fig. 2C). Full-length normal *HTT* RNA with 23 CAG triplets (FL-HTTQ23) also formed foci in a small percentage of cells, presumably as a consequence of overexpression. These results demonstrate that both truncated and FL *expHTT* RNA form structures that facilitate RNA aggregation into RNA foci, and hence that foci formation is not an artifact of a short transcript.

RNA foci are thought to be one of the major sites of interaction between RNA with repeat expansions and RBPs, including the splicing factor MBNL1^11^,^18^. Sequestration of MBNL1 by expanded CUG repeat-containing RNA foci depletes functional MBNL1 in the nucleus and induces dysregulation of alternative splicing in myotonic dystrophy type 1 (DM1)^20^,^21^. Similar co-localization of MBNL1 with expanded CUG RNA foci has been detected in SCA8^22^ and HDL2^7^. CAG foci sequestering MBNL1 have been detected in HD patient–derived fibroblasts^11^,^12^ and a *Drosophila* model of SCA3^3^,^11^,^17^.

**Figure 3:**
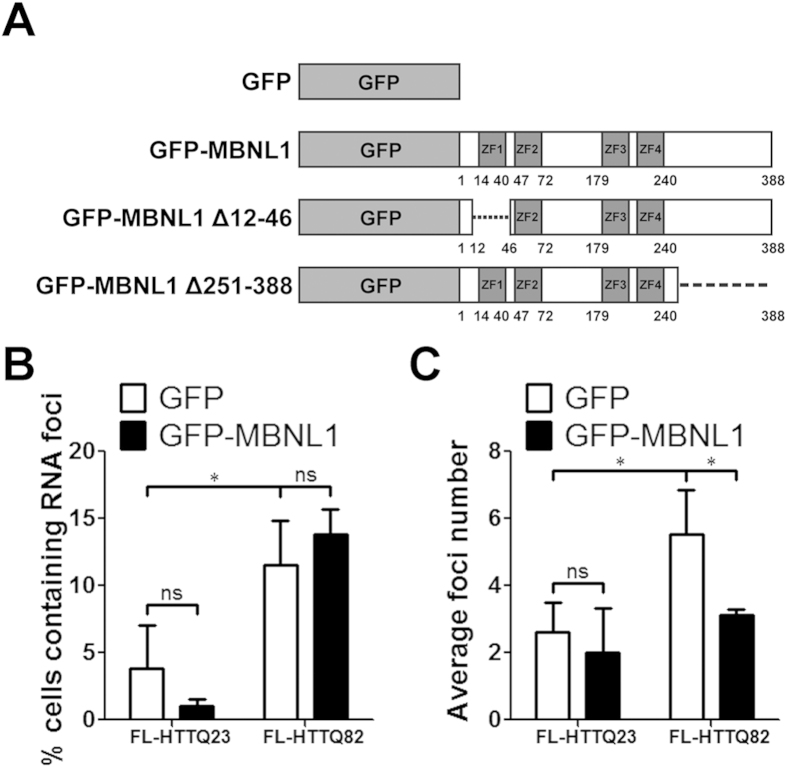
MBNL1 decreases the number of *FL-HTTQ82* RNA foci. (**A**) Schematic representation of GFP-MBNL1 plasmids. ZFs, zinc finger motifs. SK-N-MC cells were co-transfected with FL-HTT and GFP-MBNL1 plasmids for 48 hours and subjected to FISH. GFP plasmid was used as a control. Foci analysis was performed by Nikon Eclipse E400 microscopy. In each treatment, numbers of GFP-positive cells and foci-containing GFP-positive cells were counted. (**B**) Percentage of cells containing RNA foci. (**C**) Average foci number. Both experiments, two-way ANOVA, n = 3 biological replicates; *P < 0.05, **P < 0.01, ns = no significance.

To further examine the relationship between MBNL1 and *HTT* RNA foci in neuronal-like cells, we co-transfected plasmids encoding FL-HTTQ82 and either GFP or GFP-MBNL1 (Fig. 3A) into SK-N-MC cells. While multiple isoforms of MBNL1 exist, we focused on MBNL1 isoform C, also known as the 42-kD isoform, containing 388 amino acids^23^. We examined the quantitative effect of MBNL1 on foci formation of *FL-HTT* RNA. As shown in Fig. 3B, 48 hours after co-expression of a GFP control plasmid and expanded FL-HTTQ82, RNA foci were detected in ~12% of nuclei (FL-HTTQ82 + GFP). Presumably as a consequence of overexpression, normal *FL-HTTQ23* RNA also formed RNA foci, though in a much smaller subset of cells (Fig. 3B, FL-HTTQ23 + GFP). Overexpression of GFP-MBNL1 had no significant effect on the overall percentage of cells containing RNA foci generated by expression of either FL-HTTQ23 or FL-HTTQ82 (Fig. 3B, FL-HTT + GFP-MBNL1). However, GFP-MBNL1 overexpression decreased the mean number of nuclear *FL-HTTQ82* RNA foci, but not of *FL-HTTQ23* RNA foci (Fig. 3C, FL-HTTQ82 + GFP versus FL-HTTQ82 + GFP-MBNL1). Representative images of RNA foci used in these quantitative analyses are shown in Supplementary Figure S3. Therefore, while overexpression of MBNL1 does not alter the total number of cells containing *expHTT* foci, it markedly decreases the number of *expHTT* foci in each cell in which foci are detectable. No significant changes in the size of the foci were observed. This led us to hypothesize that MBNL1 changes the pool of *expHTT* available for foci formation.

### MBNL1 increases nuclear retention of FL *expHTT* RNA and decreases the expression of FL expHTT protein

**Figure 4:**
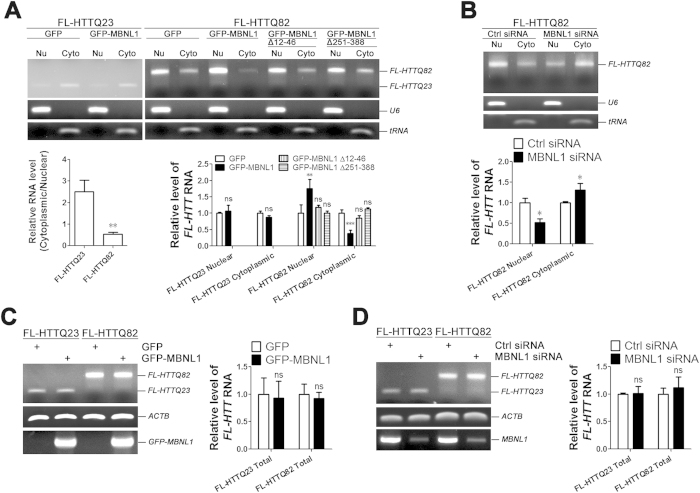
MBNL1 increases nuclear retention of expanded *FL-HTT* RNA. (**A**) Ratio of cytoplasmic to nuclear RNA levels indicates nuclear retention of *FL-HTTQ82* RNA. Student’s *t*-test, n = 3 biological replicates. **P < 0.01, versus FL-HTTQ23. Levels of cytoplasmic and nuclear *FL-HTT* RNA showed the regulatory effect of MBNL1 variant overexpression (**A**) and endogenous MBNL1 knock-down (**B**) on nuclear retention of *FL-HTTQ82* RNA. Student’s *t*-test or one-way ANOVA, n = 3 biological replicates. *P < 0.05, **P < 0.01, ***P < 0.001, ns = no significance, versus GFP group or control siRNA group. No effect of MBNL1 overexpression (**C**) or endogenous MBNL1 knock-down (**D**) on levels of total *FL-HTT* RNA was observed. Student’s *t*-test, n = 3 biological replicates. ns = no significance, versus GFP group or control siRNA group.

If foci formation results in sequestration of RNA with a consequent increase in total nuclear *expHTT* RNA, then a MBNL1-induced decrease in foci would be predicted to reduce total nuclear *expHTT* RNA. However, unexpectedly, we determined that *FL-HTTQ82* RNA is increased in the nuclear fractions of SK-N-MC cells relative to *FL-HTTQ23*, and that expression of MBNL1 further increased nuclear retention of *FL-HTTQ82* RNA by ~170% and decreased cytoplasmic *FL-HTTQ82* RNA to ~40% of control (Fig. 4A, FL-HTTQ82 + GFP-MBNL1). Knock-down of endogenous MBNL1 by siRNA decreased nuclear levels of *FL-HTTQ82* RNA by ~1.3 fold (Fig. 4B, FL-HTTQ82 + MBNL1 siRNA). Neither MBNL1 overexpression nor MBNL1 knock-down changed the total cellular level of FL *HTT* RNA (Fig. 4C,D). Together, these data suggest that nuclear retention of *expHTT* RNA is not simply a function of the formation of RNA foci, but involves other processes that are in part mediated by MBNL1.

**Figure 5:**
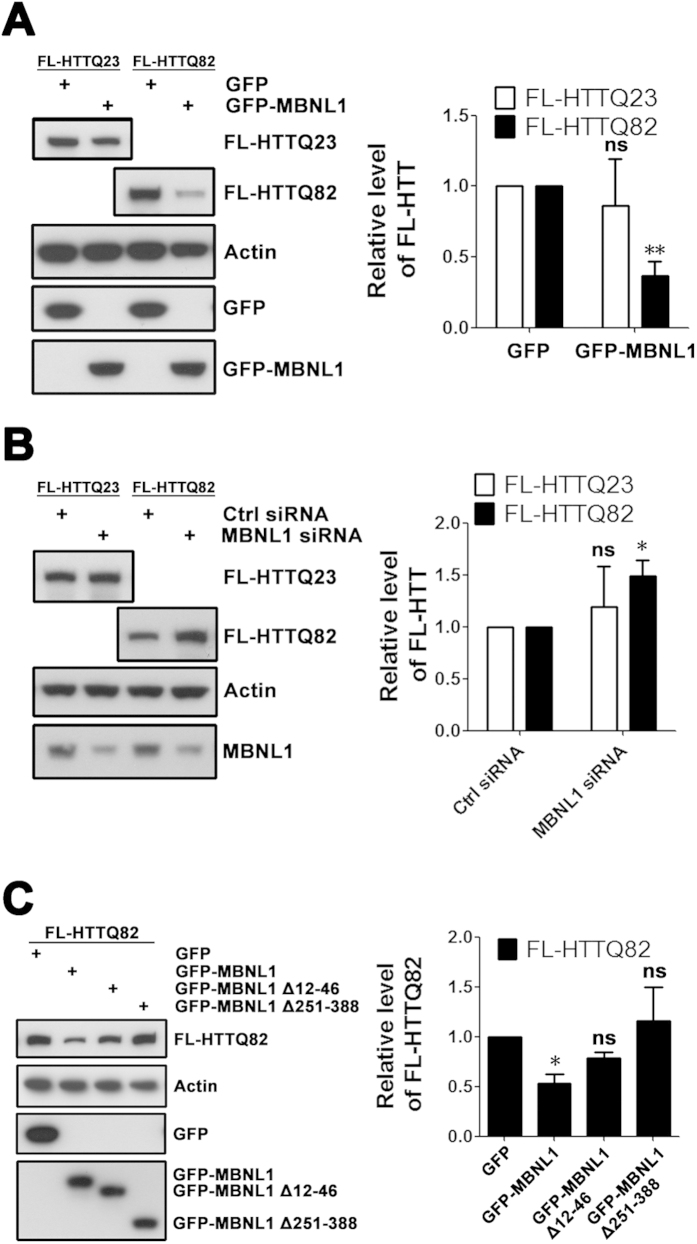
MBNL1 decreases expression of expanded FL-HTT protein. (**A**) SK-N-MC cells were co-transfected with FL-HTT and GFP-MBNL1 plasmids, and levels of FL-HTT protein were assessed by western blot 72 hours post-transfection. GFP plasmid was used as a control. Overexpression of MBNL1 decreased levels of FL-HTTQ82. Student’s *t*-test, n = 3 biological replicates. **P < 0.01, ns = no significance, versus GFP group. (**B**) SK-N-MC cells were first transfected with MBNL1 siRNA and FL-HTT plasmid. Levels of FL-HTT were assessed by western blot. Control siRNA was used as a control. Knock-down of endogenous MBNL1 increased expression of FL-HTTQ82. Student’s *t*-test, n = 3 biological replicates. *P < 0.05, ns = no significance, versus control siRNA group. (**C**) SK-N-MC cells were co-transfected with FL-HTT and GFP-MBNL1 plasmids, and levels of FL-HTT protein were assessed by western blot 72 hours post-transfection. GFP plasmid was used as control. Overexpression of GFP-MBNL1 Δ12–46 (loss of first zinc finger) and GFP-MBNL1 Δ251–388 (loss of C-terminal splicing domain) abolished the effect of MBNL1 on FL-HTTQ82 levels. One-way ANOVA, n = 3 biological replicates. *P < 0.05, ns = no significance, versus GFP group.

If MBNL1 increases nuclear retention of *expHTT* RNA, one consequence should be a decrease in the levels of the expHTT protein. We therefore assessed the levels of FL HTT protein following overexpression of MBNL1 in SK-N-MC cells. As predicted, overexpression of MBNL1 reduced the levels of FL-HTTQ82 to ~40% of control (Fig. 5A) but had no significant effect on the levels of FL-HTTQ23 (Fig. 5A). While FL HTT does not form significant protein aggregates in the SK-N-MC cell model, we observed that MBNL1 reduces both soluble and insoluble N63Q148, which readily forms protein aggregates in SK-N-MC cells (data not included). Conversely, knock-down of endogenous MBNL1 increased the levels of FL-HTTQ82 protein by ~1.5 fold, but did not have a significant effect on FL-HTTQ23 protein (Fig. 5B). We did observe variability in FL-HTTQ23 experiments, perhaps reflecting a small and inconsistent effect of MBNL1 on shorter repeats.

To further confirm that the effect of MBNL1 on nuclear retention of *expHTT* is primarily dependent on the expanded CAG repeat, we examined the effect of MBNL1 on levels of endogenous HTT, ATXN2 and ATXN3 proteins with normal repeat sizes, as well as on the expression of an exogenously expressed protein without a repeat (mRFP). MBNL1 overexpression had no significant effect on the expression of any of these proteins (Supplementary Fig. S4). Consistent with this set of observations, knock-down of MBNL1 did not change the levels of endogenous proteins with normal length repeats, and only minimally increased mRFP expression (Supplementary Fig. S4). These data indicate that the MBNL1-associated decrease in expHTT protein expression is primarily dependent on the presence of an expanded repeat.

### The MBNL1-associated increase in nuclear *expHTT* RNA depends on the RNA binding capacity and nuclear localization of MBNL1

We next sought to confirm that the MBNL1-induced increase in the level of nuclear *expHTT* RNA is dependent on the capacity of MBNL1 to bind to RNA. MBNL1 has four CCCH-type zinc finger motifs, which are all necessary for MBNL1 to bind RNA^24^,^25^. A C-terminal splicing domain includes a recently identified nuclear localization signal^26^. We therefore made two different deletions of MBNL1: a deletion of the first zinc finger (MBNL1 Δ12–46) and a deletion of the C-terminal splicing domain (MBNL1 Δ251–388, Fig. 3A). MBNL1 Δ12–46 showed an attenuated capacity to increase nuclear FL *expHTT* RNA (Figs 4A,5C). Similarly, MBNL1 Δ251–388, which, unlike MBNL1 and MBNL1 Δ12–46, is localized to both the nucleus and cytoplasm (Supplementary Fig. S5), did not significantly increase nuclear levels of *expHTT* RNA (Figs 4A,5C). These data indicate that the MBNL1-mediated increase of nuclear *expHTT* RNA requires MBNL1’s RNA-binding capacity and nuclear localization, and suggest that MBNL1 may reduce the nuclear export of expHTT.

### MBNL1 reduces non-ATG-dependent translation of an *expHTT* RNA fragment

**Figure 6:**
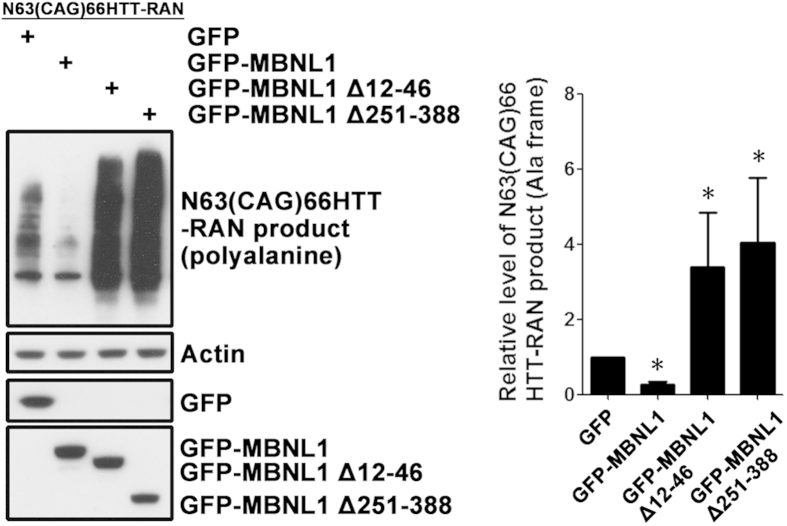
MBNL1 decreases non-ATG-initiated translation of an *expHTT* RNA fragment. SK-N-MC cells were co-transfected with N63(CAG)66HTT-RAN and GFP-MBNL1 plasmids, and 72 hours post-transfection levels of polyAla-containing protein from RAN translation were assessed by western blot. GFP plasmid was used as a control. Overexpression of MBNL1 decreased the level of polyAla RAN protein, while the MBNL1 Δ12–46 and GFP-MBNL1 Δ251–388 deletions increased the expression of polyAla-containing RAN product. One-way ANOVA, n = 3 biological replicates. *P < 0.05, versus GFP group.

*N63(CAG)66HTT* transcripts without an ATG codon produce non-ATG–translated polyAla-containing peptides in SK-N-MC cells (Supplementary Fig. S1). We next tested the effect of MBNL1 on the expression of the polyAla peptides produced by RAN translation^15^. MBNL1 decreased the expression of polyAla peptides (Fig. 6), consistent with the MBNL1-induced increase in the nuclear/cytoplasmic ratio of *expHTT*. Consistent with this result, expression of either MBNL1 Δ12–46 or MBNL1 Δ251–388 resulted in a significant increase in expression of the polyAla peptides (Fig. 6) indicating that MBNL1 that is not localized to the nucleus, or that cannot bind *expHTT*, facilitates the nuclear export and translation of *expHTT*, including RAN translation. .

### Effect of MBNL1 on *expHTT* RNA is not CAG repeat- or disease-specific

**Figure 7:**
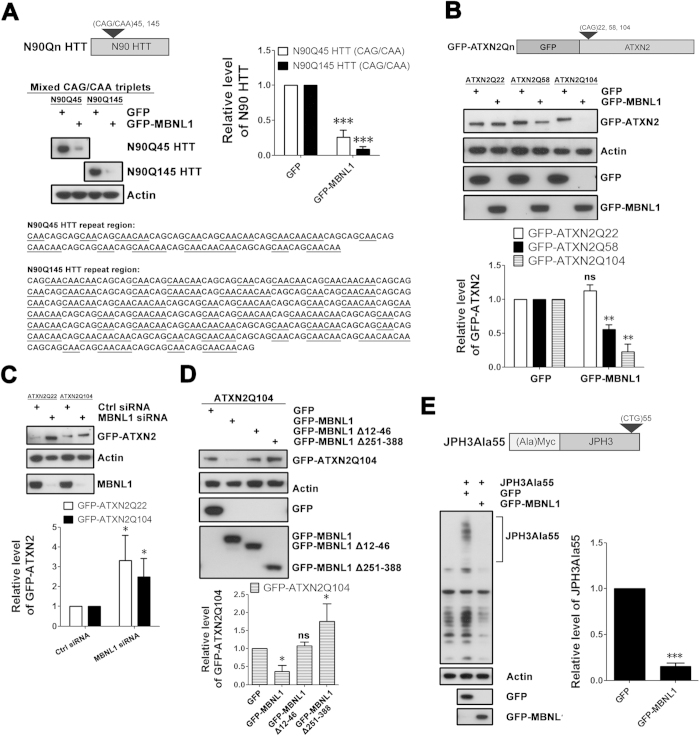
Effect of MBNL1 on *expHTT* RNA is not CAG repeat- or disease –specific. (**A**) SK-N-MC cells were co-transfected with N90QnHTT plasmid and GFP-MBNL1 plasmid and levels of N90HTT protein were assessed by western blot. GFP plasmid was used as a control. MBNL1 still decreased levels of expanded N90QnHTT encoded by CAA-interrupted CAG repeats (shown by underlines). N90Q145 HTT plasmid, like N90Q45 HTT plasmid, has heavily CAA-interrupted CAG repeats. Both experiments, Student’s *t*-test, n = 3 biological replicates. ***P < 0.001, versus GFP group. (**B**) SK-N-MC cells were co-transfected with GFP-ATXN2 and GFP-MBNL1 plasmids and levels of GFP-ATXN2 protein were assessed by western blot. GFP plasmid was used as control. Overexpression of MBNL1 decreased levels of expanded GFP-ATXN2. All experiments, student’s *t*-test, n = 3 biological replicates. *P < 0.05, **P < 0.01, ns = no significance, versus GFP group. (**C**) SK-N-MC cells were first transfected with MBNL1 siRNA and GFP-ATXN2 plasmid. Levels of GFP-ATXN2 were assessed by western blot. Control siRNA was used as a control. Knock-down of endogenous MBNL1 increased expression of GFP-ATXN2. Student’s *t*-test, n = 3 biological replicates. *P < 0.05, versus control siRNA group. (**D**) SK-N-MC cells were co-transfected with GFP-ATXN2 and GFP-MBNL1 plasmids, and levels of GFP-ATXN2 protein were assessed by western blot 72 hours post-transfection. GFP plasmid was used as a control. Overexpression of GFP-MBNL1 Δ12–46 (loss of first zinc finger) and GFP-MBNL1 Δ251–388 (loss of C-terminal splicing domain) abolished the effect of MBNL1 on GFP-ATXN2Q104 levels. One-way ANOVA, n = 3 biological replicates. *P < 0.05, ns = no significance, versus GFP group. (**E**) SK-N-MC cells were co-transfected with JPH3Ala55 and GFP-MBNL1 plasmids and levels of JPH3Ala55 protein were assessed by western blot 72 hours pos-transfection. GFP plasmid was used as a control. Overexpression of MBNL1 decreased the levels of JPH3Ala55, encoded by expanded CUG repeats. Student’s *t*-test, n = 3 biological replicates. ***P < 0.001, versus GFP group.

The capacity of MBNL1 to bind CAG repeats *in vitro* may depend on the RNA hairpin structure formed by strong C-G parings^27^,^28^. To indirectly test this possibility in our cell model, we modified HTT constructs N90Q45 and N90Q145 (each encoding the first 90 amino acids in HTT) by interrupting CAG triplets with CAA triplets (as depicted in Fig. 7A). Addition of MBNL1 resulted in decreased expression of these interrupted constructs (Fig. 7A), demonstrating that the effect of MBNL1 on *expHTT* is not dependent on a pure CAG repeat.

HD is one of nine autosomal dominant neurodegenerative diseases caused by CAG repeat expansions^29^. A CAG/CTG repeat expansion in *ATXN2* causes spinocerebellar ataxia type 2 (SCA2)^30^. To test if the effect of MBNL1 is disease-specific, we co-expressed MBNL1 with a FL GFP-tagged ataxin-2 (ATXN2) construct. GFP-MBNL1 overexpression significantly decreased the expression of expanded ATXN2Q58 and ATXN2Q104 protein to 50% and 20% of control, respectively (Fig. 7B). Conversely, MBNL1 knock-down increased expression of expanded ATXN2Q104 by ~2.5 fold (Fig. 7C). ATXN2Q22 was also up-regulated by MBNL1 knock-down (Fig. 7C). Interestingly, MBNL1 ∆12–46 had no significant effect on the level of expanded ATXN2Q104, while MBNL1 ∆251–328 significantly increased expanded ATXN2Q104 expression (Fig. 7D). We conclude that the effect of MBNL1 on protein expression of transcripts with CAG repeats, and particularly on expanded repeats, is not limited to *expHTT*. However, the degree to which MBNL1 binds to different CAG repeat–containing transcripts may be influenced by disease-specific sequences flanking the CAG repeats.

We next sought to determine if the effect of MBNL1 on translation is CAG repeat-specific. CUG repeat expansion in *JPH3* causes HDL2, in part via RNA-mediated neurotoxicity^7^,^31^. Alternative splicing of *JPH3* results in transcript variants in which the CUG repeat resides in the 3′ UTR or within open reading frames translated into polyAla or polyleucine^32^. We expressed a *JPH3* construct in which the repeat is in-frame for translation into polyAla (JPH3Ala55)^32^ with and without MBNL1 and observed that MBNL1 decreased the expression of JPH3Ala55 protein (Fig. 7E). This experiment suggests that the effect of MBNL1 on the expression of proteins derived from transcripts with expanded repeats is not CAG repeat-specific.

### U2AF65 stimulates nucleocytoplasmic export of FL *expHTT* RNA

**Figure 8:**
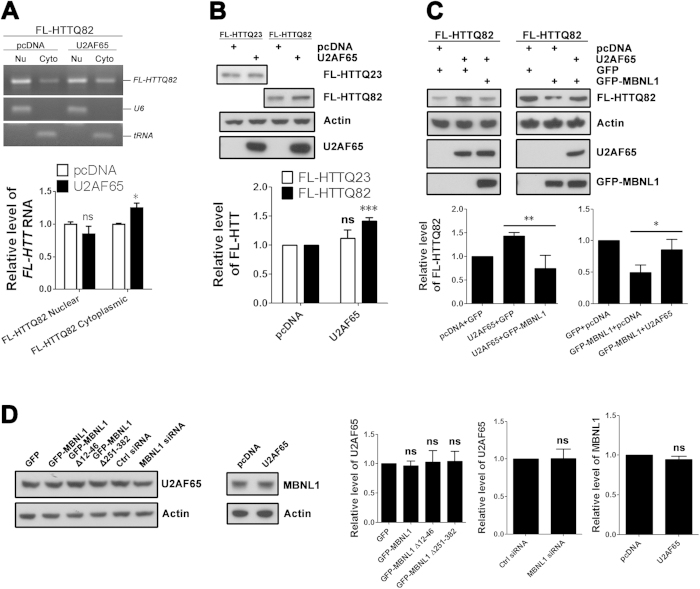
U2AF65 competes with MBNL1 in the export of expanded *FL-HTT* RNA. (**A**) SK-N-MC cells were co-transfected with FL-HTTQ82 and U2AF65 plasmids. The cytoplasmic and nuclear RNA fractions were extracted and examined by RT-PCR 48 hours post-transfection. pcDNA3.1 plasmid was used as a control. Overexpression of U2AF65 increased the export of *FL-HTTQ82* RNA. Student *t*-test, n = 3 biological replicates. *P < 0.05, ns = no significance, versus pcDNA3.1 group. (**B**) SK-N-MC cells were co-transfected with FL-HTT and U2AF65 plasmids, and levels of FL-HTT protein were assessed by western blot 72 hours post-transfection. pcDNA3.1 plasmid was used as control. Overexpression of U2AF65 increased the levels of FL-HTTQ82 protein. Student’s *t*-test, n = 3 biological replicates. ***P < 0.001, ns = no significance, versus pcDNA3.1 group. (**C**) SK-N-MC cells were triple-transfected with FL-HTTQ82, GFP-MBNL1 and U2AF65 plasmids, and levels of FL-HTTQ82 protein were assessed by western blot 72 hours post-transfection. GFP and pcDNA3.1 plasmids were used as controls, respectively. Overexpression of MBNL1 reversed the effect of U2AF65 on the level of FL-HTTQ82 and vice versa. Both experiments, one-way ANOVA, n = 3 biological replicates. *P < 0.05, **P < 0.01. (**D**) SK-N-MC cells were transfected with GFP-MBNL1 plasmids and MBNL1 siRNA, or U2AF65 plasmid and levels of endogenous U2AF65 and MBNL1, respectively, were examined by western blot. GFP plasmid, control siRNA and pcDNA3.1 plasmid, respectively, were used as controls. Overexpression of MBNL1 had no effect on endogenous levels of U2AF65 and vice versa. Knock-down of endogenous MBNL1 had no effect of endogenous levels of U2AF65. All experiments, Student’s *t*-test or one-way ANOVA, n = 3 biological replicates. ns = no significance, versus GFP group, control siRNA group and pcDNA3.1 group, respectively.

Our data so far support the idea that MBNL1 increases nuclear retention of *expHTT* RNA and other transcripts with expanded repeats by interfering with nuclear export processes. The nuclear export of mRNA is mainly mediated by the nuclear export factor 1 (NXF1) receptor pathway^33^, which was recently implicated in the nuclear retention of expanded *ATXN3* RNA in a fly model of SCA3^34^. In this model the protein U2AF65 interacts with expanded CAG repeat-containing RNA and serves as an adaptor to link the transcript to NXF1. The same export mechanism may be disrupted in HD^34^. Interestingly, U2AF65 and MBNL1 were previously identified as splicing factors that compete with each other at a *cardiac troponin T* (*cTNT*) splicing site^35^. To determine if the NXF1 pathway is involved in the nuclear export of *expHTT*, we overexpressed U2AF65 with FL expHTT. In SK-N-MC cells, overexpression of U2AF65 increases the levels of expHTT protein (Fig. 8B), opposite to the effect observed with MBNL1. We therefore speculated that nuclear retention of *expHTT* RNA in HD may be triggered by an aberrant interaction of *expHTT* with MBNL1, with a consequent loss of U2AF65 binding and a disruption of NFX1 pathway-mediated export of *expHTT* RNA. Consistent with this speculation, co-expression of MBNL1 blocked the effect of U2AF65 on FL-HTTQ82 expression (Fig. 8C). The effect is unlikely to derive from an artifact of construct expression levels, as U2AF65 and MBNL1 do not appear to influence the endogenous expression of each other (Fig. 8D).

## Discussion

There is growing evidence that mutant RNA contributes to the pathogenesis of multiple repeat expansion diseases^3^,^7^,^8^,^20^,^22^. Our data provide further support for a role of *expHTT* RNA in HD pathogenesis by demonstrating the cytotoxicity of both truncated non-translatable and RAN-translated *expHTT* RNA fragments containing expanded CAG repeats of different lengths (Fig. 1). Future model systems that use FL constructs and automated longitudinal monitoring of toxic damage to individual cells as developed by Finkbeiner and colleagues^36^, will help refine the quantitative and temporal relationship of RNA and protein toxicity in specific cell types.

Several HD mouse models have been used to demonstrate that alterations in protein sequence markedly suppress the phenotype of HD, including the YAC128 transgenic mice “short stop” (a mutation prevents expression of full length protein), the YAC128 line in which the caspase -6 cleavage site has been eliminated, and the BAC transgenic mice expressing full-length mutant huntingtin with serines 13 and 16 mutated to aspartate^37^,^38^,^39^. The lack of phenotype in these mice supports the argument that mutant protein, and not *expHTT* RNA, is essential to disease pathogenesis. In addition, the BACHD mouse model^40^, in which the expanded repeat is interrupted ([CAACAGCAGCAACAGCAA]n), reproduces many of the features of HD, leading to the speculation that the interruptions in the repeat prevent the formation of abnormal RNA structures, hence eliminating a contribution of RNA toxicity. However, it is difficult to reconcile this conclusion with the increasingly compelling data, primarily from cell models (including human HD cells), that RNA is also important in disease pathogenesis. Part of the problem is that mouse models of repeat disease are inherently imperfect—each defers from other mouse models, from cell models, and from human disease in the degree to which they recapitulate the mechanistic complexity of repeat diseases, including variables such as repeat length and integrity, transcript and protein expression levels, localization and aggregation of protein and RNA, and the presence of RAN translation^7^,^9^,^10^,^11^,^12^,^13^,^14^,^15^,^32^,^34^. It is not possible to evaluate the role of RNA toxicity in cell or mouse models without data on expression levels, sub-cellular distribution, and aggregation status of *expHTT* RNA. Finally, it is quite possible that longer repeats shift the relevant importance of RNA and protein toxicity, so that the long repeats used in most mouse models may obscure the role of RNA toxicity.

In further support for the role of RNA in HD, we show that truncated *HTT* RNA aggregates into RNA foci in neuronal-like cells and in neurons of an HD transgenic mouse model (Supplementary Fig. S2). We also show for the first time that FL *expHTT* RNA readily forms RNA foci in neuronal-like cells (Fig. 2). In addition, we show that co-expression of MBNL1 decreases the number of RNA foci formed from exogenous *expHTT* RNA (Fig. 3). The effect of MBNL1 on the *expHTT* RNA foci formation is opposite to recent data suggesting that an exon 1 *expHTT* fragment forms RNA foci only when co-expressed with MBNL1^26^. We posit that this discrepancy is primarily due to the sensitivity of our assay in detecting RNA foci formed by both truncated and FL *expHTT* transcripts; a difference in cell types may also contribute to the different findings.

Our data demonstrate that FL *expHTT* RNA is retained in the nucleus of neuronal cells, in agreement with evidence of nuclear retention of *HTT* RNA in the R6/2 mouse model of HD^34^ and our own data, and those of others, showing that nuclear retention of *expHTT* fragments is increased by MBNL1^26^. Counterintuitively, we determined that the retention is not due to *expHTT* RNA aggregation into RNA foci, but involves aberrant interaction between FL *expHTT* RNA and MBNL1 (Fig. 4).

Our evidence that neither knock-down nor overexpression of MBNL1 has a significant effect on the total levels of FL *HTT* RNA (Fig. 4) differs from *Drosophila* models of SCA3^3^ and myotonic dystrophy 2 (DM2)^41^. While the MBNL1 effect on total RNA levels may be specific for the fly models, it is also possible that the effect of MBNL1 on localization and/or levels of expRNA is dependent on the sequence flanking the repeat.

Overexpression of MBNL1 decreases non-ATG-initiated translation, while both MBNL1 Δ12–46 and MBNL1 Δ251–388 significantly facilitate this translational mechanism (Fig. 6). One possible explanation is that MBNL1 Δ12–46 inefficiently binds to *expHTT* RNA. While this is not sufficient to trigger nuclear retention, the imposed structural changes on the *expHTT* RNA may facilitate RAN translation of *expHTT* RNA. On the other hand, MBNL1 Δ251–388, which has full RNA-binding ability, may be insufficiently retained in the nucleus to induce nuclear retention of *expHTT R*NA (Supplementary Fig. S5), while in the cytoplasm it may bind to *expHTT* RNA and facilitate non-ATG–dependent translation. These effects of MBNL1 variants on RAN translation may provide useful clues to understanding RAN translation.

U2AF65, a splicing factor and a component of the NXF1 receptor export pathway^42^, stimulates nuclear export and increases the translation of *expHTT* RNA (Fig. 8), opposite to the effect of MBNL1. A potential explanation is that increased binding of MBNL1 to *expHTT* RNA also results in sequestration of U2AF65, disrupting the assembly of the NXF1 complex and leading to nuclear retention of *expHTT* RNA. Alternatively, and with more specificity, MBNL1 may interfere with the action of U2AF65 on *expHTT*, altering the entry of *expHTT* into the export pathway. While increasing U2AF65 levels is unlikely to decrease the toxicity of expHTT (Supplementary Fig. S6), further characterization of the *expHTT* RNA export pathway may identify novel targets with therapeutic potential. Intriguingly, the NXF1 receptor pathway was recently linked to DM1 by the finding that Aly/REF is associated with nuclear accumulation of expanded *DMPK* transcripts^43^. Insertion of a WPRE (woodchuck post-transcriptional regulatory element) at the 3′-end of the *expDMPK* 3′-UTR stimulated the export of expanded *DMPK* RNA and rescued muscle cell differentiation^44^. In addition, increasing Staufen1, a double-stranded RNA (dsRNA)-binding protein implicated in multiple post-transcriptional gene-regulatory processes^45^, and thereby increasing nuclear export, rescued three hallmarks of DM1 pathology (aberrant splicing, nuclear export, and translation of *expCUG* RNA)^46^. A concern is that increasing nuclear export could elevate the levels of expHTT protein and increase neurotoxicity. However, if RNA toxicity is reduced, cells may more efficiently degrade or otherwise protect themselves from expHTT protein, consistent with the recognition that expHTT protein turnover is 3-fold more important for neuronal survival than is protein level^47^. The net effect, we speculate, would be a decrease in neuronal toxicity. Targeting the interactions of MBNL1 and U2AF65 with *expHTT* with antisense oligonucleotides or small molecules may therefore have substantial therapeutic benefits^48^,^49^,^50^.

Analysis of the effect of MBNL1 on the effect of expHTT toxicity is complicated by the finding that with or without the N-terminal zinc finger domain or NLS signal, MBNL1 itself increases cytotoxicity (Supplementary Fig. S6), and MBNL1 knockdown decreases SK-N-MC cell proliferation (Supplementary Fig. S6). Similarly, overexpression of U2AF65 reduced proliferation of SK-N-MC cells (Supplementary Fig. S6), complicating the interpretation of the apparent effect of U2AF65 on reducing expHTT toxicity. This is not unexpected as both MBNL1 and U2AF65 have multiple functions^26^,^34^,^51^,^52^,^53^,^54^ and any changes in the levels of RBPs are likely to disrupt multiple cellular pathways. Indeed, the effects of MBNL1 overexpression on models of repeat expansion disease appear to vary as a function of the model organism, the repeat expansion under exploration, and levels of transcript expression^55^,^56^,^57^,^58^. We speculate that the effects of MBNL1 on missplicing and nuclear retention exist in homeostatic tension, and that disruption of the homeostasis, whether through overexpression or knockdown, can result in neurotoxicity.

Taken together, our data provide mechanistic support for the hypothesis that *expHTT* transcripts contribute to the pathogenesis of HD and support an examination of nuclear export pathways as a source of novel therapeutic targets for HD.

## Materials and Methods

### Reagents, cells and mice

Control siRNA (sc-37007) and human MBNL1 siRNA (sc-60988) were purchased from Santa Cruz Biotechnology (Santa Cruz, CA). The antibodies used for immunoblotting were as follows: anti-huntingtin (MAB2166, 1:1000), anti-polyglutamine, 1C2 (MAB1574, 1:2000), anti-ATXN3 (MAB5360, 1:500) from EMD Millipore (Billerica, MA); anti-ATXN2 (611378, 1:500) from BD Transduction Laboratories (San Jose, CA); anti-β-actin (ab8224, 1:5000) from Abcam (Cambridge, MA); anti-GFP (G10362, 1:2000), anti-myc (AHO0062, 1:2000), anti-HA (32–6700, 1:1000) from Life Technologies (Grand Island, NY); anti-MBNL1 (A2764, 1:1000) from Dr. Charles A. Thornton (University of Rochester, Rochester, NY); anti-mRFP (5F8, 1:1000) from Chromotek (Martinsried, Germany); anti-N-terminus of huntingtin (sc-8767, 1:200) from Santa Cruz Biotechnology; anti-FLAG (4C5, 1:1000) from Origene (Rockville, MD).

Neuroblastoma cell lines SK-N-MC (HTB-10; ATCC) and SH-SY5Y (CRL-2266; ATCC) were cultured in Dulbecco’s Modified Eagle Medium with 4.5 g/L glucose and supplemented with 10% fetal bovine serum. Nine-month-old C57BL/6J wild type mice were from The Jackson Laboratory (Bar Harbor, ME), and N586-82Q transgenic HD mice were previously described^19^. The breeding and subsequent use of mice were approved by the ACUC of Johns Hopkins University, Baltimore, MD. Mice were housed at the East Baltimore campus rodent vivarium and maintained on a standard circadian cycle with free access to water and standard chow.

### Plasmid construction

Plasmids encoding truncated N-terminal HTT (N63QnHTT) and FL HTT (FL-HTTQn) were described by Cooper *et al.* and Ratovitski *et al.*, respectively^59^,^60^. N90QnHTT plasmids were obtained from Coriell Institute for Medical Research (Camden, NJ). The translation start codon of N63QnHTT plasmids was deleted and a TAG stop codon was introduced immediately 5′ to the repeat to produce N63(CAG)nHTT plasmids. To examine RAN translation, *N63(CAG)nHTT* inserts were PCR-amplified and cloned into the XhoI and XbaI sites of the A8(*KKQ_EXP_)-3Tf1 vector to produce N63(CAG)nHTT-RAN plasmids^15^. JPH3Ala55 plasmid expressing the *JPH3* transcript in the polyalanine frame and JPH3-(CTG)55 plasmid expressing a non-translatable *JPH3* transcript were described by Seixas *et al.*^32^. GFP-MBNL1 plasmid expressing GFP-tagged human MBNL1 isoform C was previously described by Lin *et al.*^23^. GFP-MBNL1 Δ12–46 was constructed by deleting amino acids 12–46 of MBNL1 using primers 5′-CAC CAA TTC GGG ACA CAA ATG GAC GAG TAA TCG C-3′ (forward) and 5′-GCG ATT ACT CGT CCA TTT GTG TCC CGA ATT GGT G-3′ (reverse) and the QuikChange II XL Site-Directed Mutagenesis Kit (Agilent Technologies, Santa Clara, CA). GFP-MBNL1 Δ251–388 was constructed by mutating a CAA codon to a TAA stop codon at amino acid 251 using primers 5′-ATC AAG GCT GCC TAA TAC CAG GTC A-3′ (forward) and 5′-TGA CCT GGT ATT AGG CAG CCT TGA T-3′ (reverse) and the QuikChange II XL Site-Directed Mutagenesis Kit. pcDNA3.1 and pcDNA-mRFP plasmids were from Life Technologies. eEF1A1-Myc-FLAG and AKT-HA plasmids encoding myc- and FLAG-tagged eEF1A1 and HA-tagged AKT, respectively, were from Origene. U2AF65 and GFP-ATXN2Qn plasmids were previously described^30^,^34^. All plasmids were confirmed by sequencing before use.

### Fluorescence *in situ* hybridization and microscopy

SK-N-MC cells were transfected with plasmids expressing FL *HTT* transcripts and fixed in 4% paraformaldehyde 48 hours post-transfection. Frontal cortices from wild-type or N586-82Q HD mice were collected and frozen-sectioned. Fluorescence *in situ* hybridization (FISH) with or without DNase/RNase treatment was performed as previously described^7^, and images were taken using an LSM 510 confocal microscope (Zeiss, Thornwood, NY) or Nikon Eclipse E400 epifluorescence microscope (Nikon Instruments Inc., Melville, NY).

### RNA foci analysis

SK-N-MC cells were co-transfected with FL-HTT and GFP-MBNL1 plasmids, and 48 hours after transfection FISH analysis was performed. Twenty-five fields per treatment were randomly selected. In each view, the number of GFP-positive cells, number of RNA foci-containing GFP-positive cells and number of RNA foci in each cell were manually counted in a single-blind manner. The percentage of cells containing foci was calculated as [100*number of foci-containing GFP-positive cells/number of GFP-positive cells]. Average foci number was calculated as [number of RNA foci/number of foci-containing GFP-positive cells].

### RNA fractionation, RNA extraction and RT-PCR

SK-N-MC cells were transfected with indicated plasmids and, 48 hours post-transfection, total RNA was extracted by TRIZOL reagent (Life Technologies). Nucleocytoplasmic fractionation of RNA was performed as previously described^34^ with some modifications. Briefly, cells were washed with wash buffer (10 mM Tris-HCl, 140 mM NaCl, 1.5 mM MgCl_2_, 10 mM EDTA, pH 7.4) and lysed on ice in lysis buffer (10 mM Tris-HCl, 140 mM NaCl, 1.5 mM MgCl_2_, 10 mM EDTA, 0.5% Triton X-100, 40 U/ml RNasin, pH 7.4) for 5 minutes. After centrifugation at 12000 × g for 5 minutes, the supernatants containing the cytoplasmic fraction of RNA were collected. The remaining nuclear pellets were rinsed with lysis buffer twice and finally centrifuged as the nuclear fraction. Cytoplasmic and nuclear RNA were then extracted by TRIZOL reagent. One μg of RNA was used to synthesize cDNA by the SuperScript III First-Strand Synthesis System (Life Technologies). PCR reactions were performed using the following primers: *N63HTT* forward 5′-GGG CCC TTC GAA CAA AAA CTC​-3′, reverse 5′-TAG AAG GCA CAG TCG AGG-3′; *FL-HTT* forward 5′-AAT ACG ACT CAC TAT AGG G-3′, reverse 5′-CTT TCT TTG GTC GGT GCA GCG-3′; *U6* forward 5′-GTG CTC GCT TCG GCA GCA CAT ATA C-3′, reverse 5′-AAA AAT ATG GAA CGC TTC ACG AAT TTG-3′; *tRNA* forward 5′-AGC AGA GTG GCG CAG CGG-3′, reverse 5′-GAT CCA TCG ACC TCT GGG TTA-3′; *ACTB* forward 5′-ATG TGC AAG GCC GGC TTC GC-3′, reverse 5′-CCA CAC GCA GCT CAT TGT AG-3′; *GFP-MBNL1* forward 5′-CAT GGT CCT GCT GGA GTT CGT G-3′, reverse 5′-TTG TGG CTA GTC AGA TGT TCG-3′; endogenous *MBNL1* forward 5′-ATT ACA ACC CGT GCC AAT GT-3′, reverse 5′-TTG TGG CTA GTC AGA TGT TCG-3′.

### Western blots

SK-N-MC cells were plated in 6-well plates (200,000 cells/well), transfected with 4 μg of plasmids and 4 μL of Lipofectamine 2000 (Life Technologies), and protein extracts were analyzed by immunoblotting 72 hours post-transfection. For knock-down of MBNL1, cells were first transfected with 200 pmol of siRNA. After 72 hours cells were further transfected with 100 pmol of siRNA plus 2 μg of plasmids and analyzed by immunoblotting 48 hours post-transfection. Cells were lysed with RIPA buffer (Sigma-Aldrich, St. Louis, MO), and the protein lysates were subjected to SDS-polyacrylamide gel electrophoresis (SDS-PAGE) and transferred to nitrocellulose membranes. Membranes were blocked, probed with primary antibody at 4 °C overnight, washed, and incubated with HRP-conjugated secondary antibodies, and the proteins were then visualized using the ECL Prime Western Blotting System (GE Healthcare, UK) and Hyperfilm ECL (GE Healthcare).

### Caspase activity assay

SK-N-MC and SH-SY5Y cells were plated in 96-well plates (2,500 cells/well) and transfected with 0.2 μg of plasmids and 0.2 μL of Lipofectamine 2000. Cytotoxicity was assessed at 72 hours post-transfection by measuring caspase-3/7 activities using the Caspase-Glo 3/7 Assay system (Promega, Madison, WI).

### MTT proliferation assay

SK-N-MC cells were plated in 96-well plate (5,000 cells/well) and transfected with 0.2 μg of plasmids and 0.2 μL of Lipofectamine 2000. Cell numbers before and after transfection were assessed 72 hours post-transfection using the Vybrant MTT Cell Proliferation Assay Kit (Life Technologies).

### Statistical analysis

At least three biological replicates of each experiment were performed. Data were presented as mean ± SD. The results were analyzed using Student’s t-test, one-way analysis of variance (ANOVA) followed by Dunnett’s or Bonferroni’s post-hoc test, or two-way ANOVA followed by Bonferroni’s post-hoc test. Statistical significance was set at P value < 0.05.

## Additional Information

**How to cite this article**: Sun, X. *et al.* Nuclear retention of full-length *HTT* RNA is mediated by splicing factors MBNL1 and U2AF65. *Sci. Rep.* **5**, 12521; doi: 10.1038/srep12521 (2015).

## Supplementary Material

Supplementary Information
