# Supplementary material for: Nuclear retention of full-length HTT RNA is mediated by splicing factors MBNL1 and U2AF65
Source: Sci Rep. 2015 Jul 28;5:12521. doi: 10.1038/srep12521 (PMC4517393; doi:10.1038/srep12521)
Supplement: Supplementary Information [file srep12521-s1.doc]

**Nuclear retention of full-length *HTT* RNA is mediated by splicing factors MBNL1 and U2AF65**

Xin Sun^1, 7^, Pan P. Li^1^, Shanshan Zhu^1^, Rachael Cohen^1^, Leonard O. Marque^1^, Christopher A. Ross^1, 2, 3, 4^, Stefan M. Pulst^5^, Ho Yin Edwin Chan^6^, Russell L. Margolis^1, 2, 4^ & Dobrila D. Rudnicki^1, 4^

^1^Department of Psychiatry and Behavioral Sciences, Division of Neurobiology, ^2^Department of Neurology, ^3^Department of Neuroscience, and ^4^Program of Cellular and Molecular Medicine, Johns Hopkins University School of Medicine, Baltimore, Maryland, USA

^5^Department of Neurology, University of Utah, Salt Lake City, Utah, USA

^6^Laboratory of Drosophila Research, School of Life Sciences, Faculty of Science, The Chinese University of Hong Kong, Shatin, N.T., Hong Kong SAR, China

^7^Guangdong-Hong Kong-Macau Institute of CNS Regeneration, Jinan University, Guangzhou, Guangdong, China

**Supplementary Figures**

**
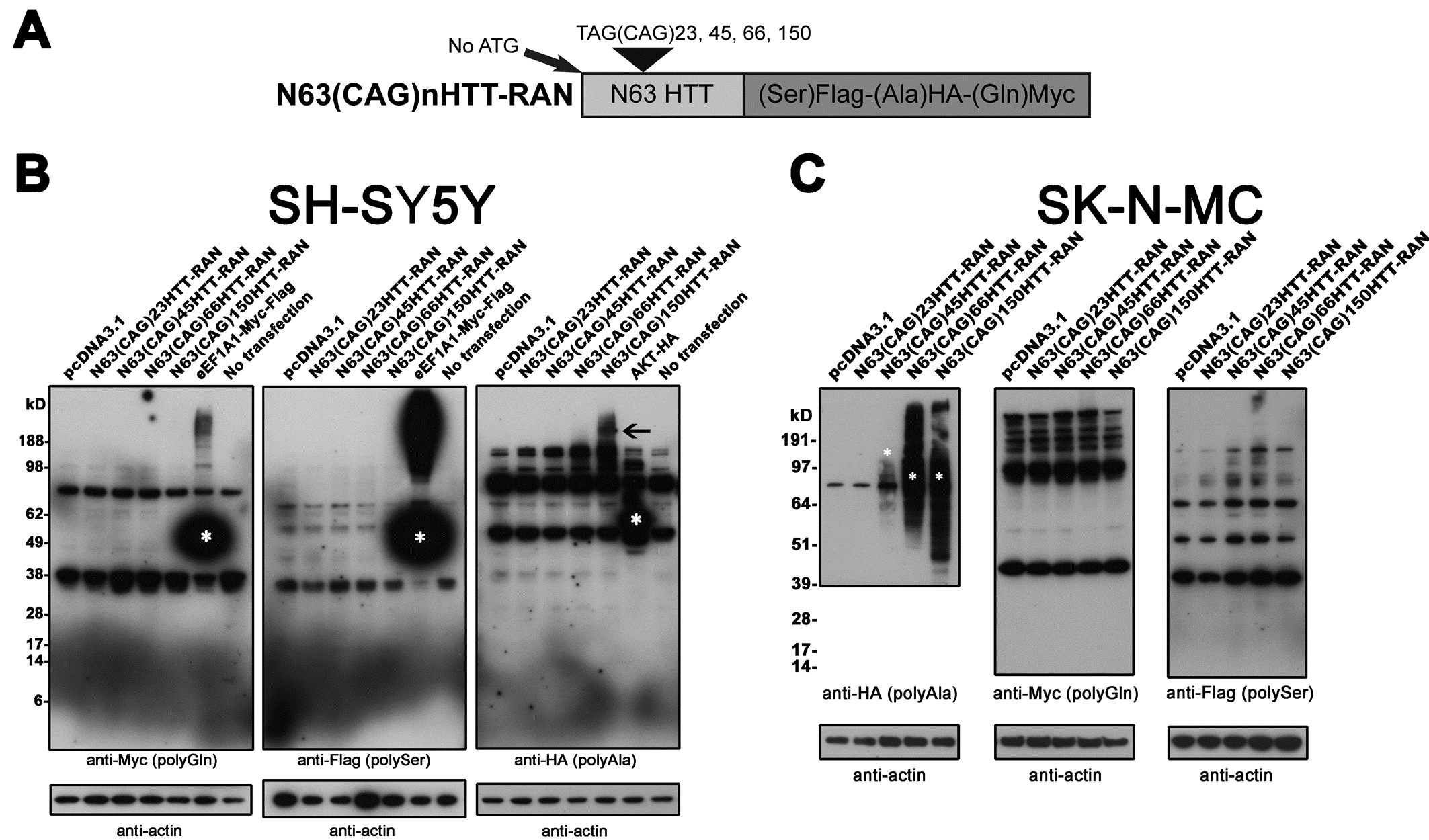
**

**Fig. S1 RAN translation of *expHTT* RNA.** (**A**) Schematic representation of *N63(CAG)nHTT*-RAN plasmids used to evaluate the presence of RAN translation products in cells expressing *HTT* RNA. (**B**) SH-SY5Y cells were transfected with *N63(CAG)nHTT*-RAN plasmids, and the presence of RAN translation was assessed by western blot 72 hours post-transfection. pcDNA3.1 plasmid was used as a negative control. eEF1A1-Myc-Flag and AKT-HA plasmids were used as positive controls for antibodies used in the experiment (asterisks). Signals were recorded after at least 30 minutes of film exposure. PolyAla-containg protein was detected in cells expressing *N63(CAG)150HTT*-RAN plasmid (arrow). (**C**) SK-N-MC cells were subject to the same transfection. Seventy-two hours after transfection, polyAla-containing peptides were detected in cells expressing from 45 to 150 CAG repeats (asterisks). Cells transfected with *N63(CAG)66HTT*-RAN constructs express minimal levels of polyGln and polySer-containing proteins.

**
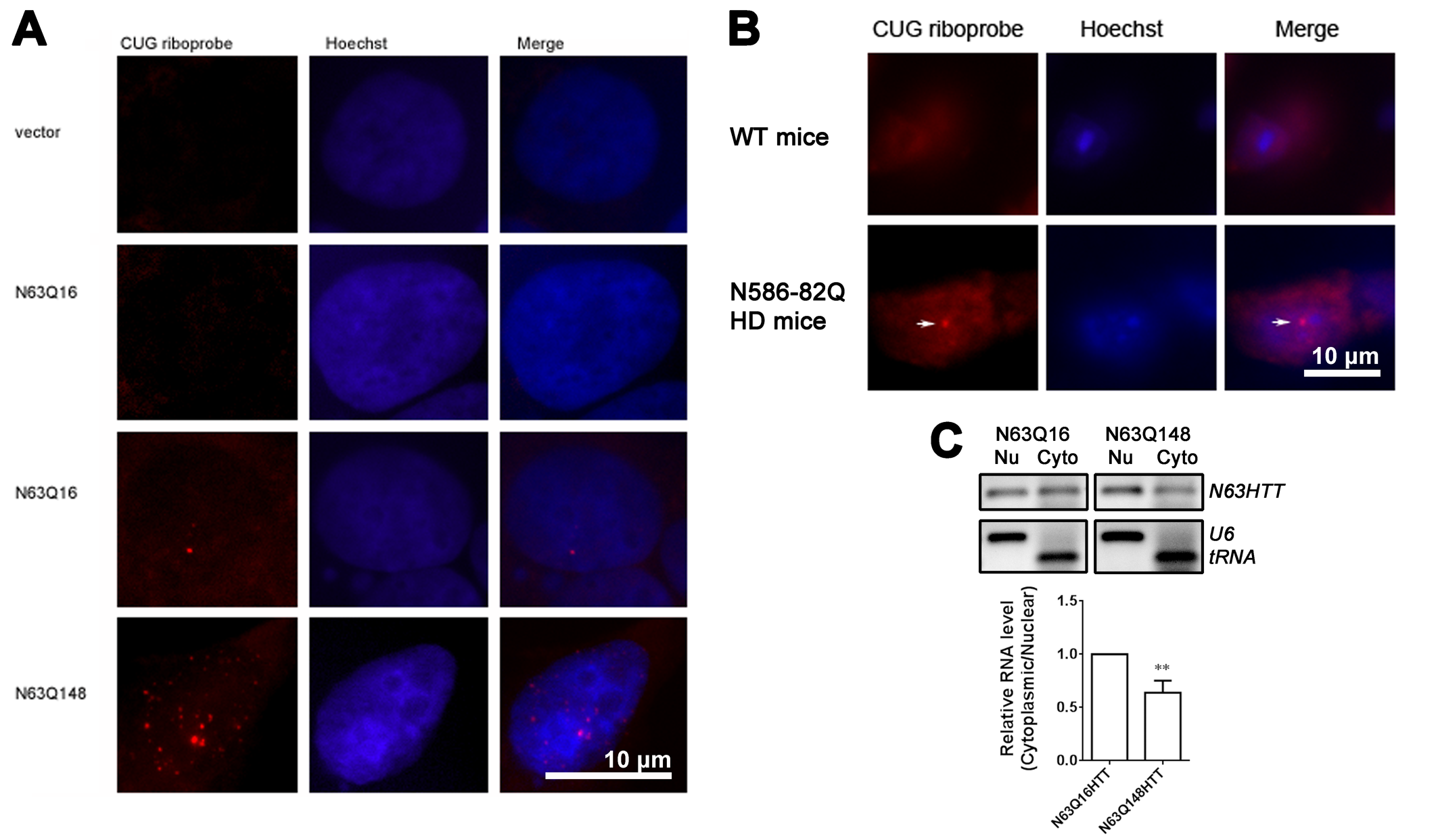
**

**Fig. S2 RNA foci formation and nuclear retention of truncated *HTT* transcripts in a cell and a transgenic mouse model.** (**A**) Truncated *N63HTT* RNA forms RNA foci in SK-N-MC cells, as determined by FISH with a CUG20 riboprobe. (**B**) RNA foci are present in the frontal cortex of the N586-82Q transgenic HD mouse model (red, arrow), but not in wild-type mice. Hoechst was used as a nuclear marker. Scale bar, 10 μm. (**C**) SK-N-MC cells were transfected with *N63HTT* plasmids, and 48 hours post-transfection cytoplasmic and nuclear RNA fractions were isolated and examined by RT-PCR. Levels of *tRNA-met* and *U6* transcripts were used as loading controls of cytoplasmic and nuclear RNA, respectively. Ratios of cytoplasmic to nuclear RNA levels indicated nuclear retention of *N63Q148HTT* RNA. Student’s *t*-test, n=3 biological replicates. **P<0.01, versus *N63Q16HTT*.

**
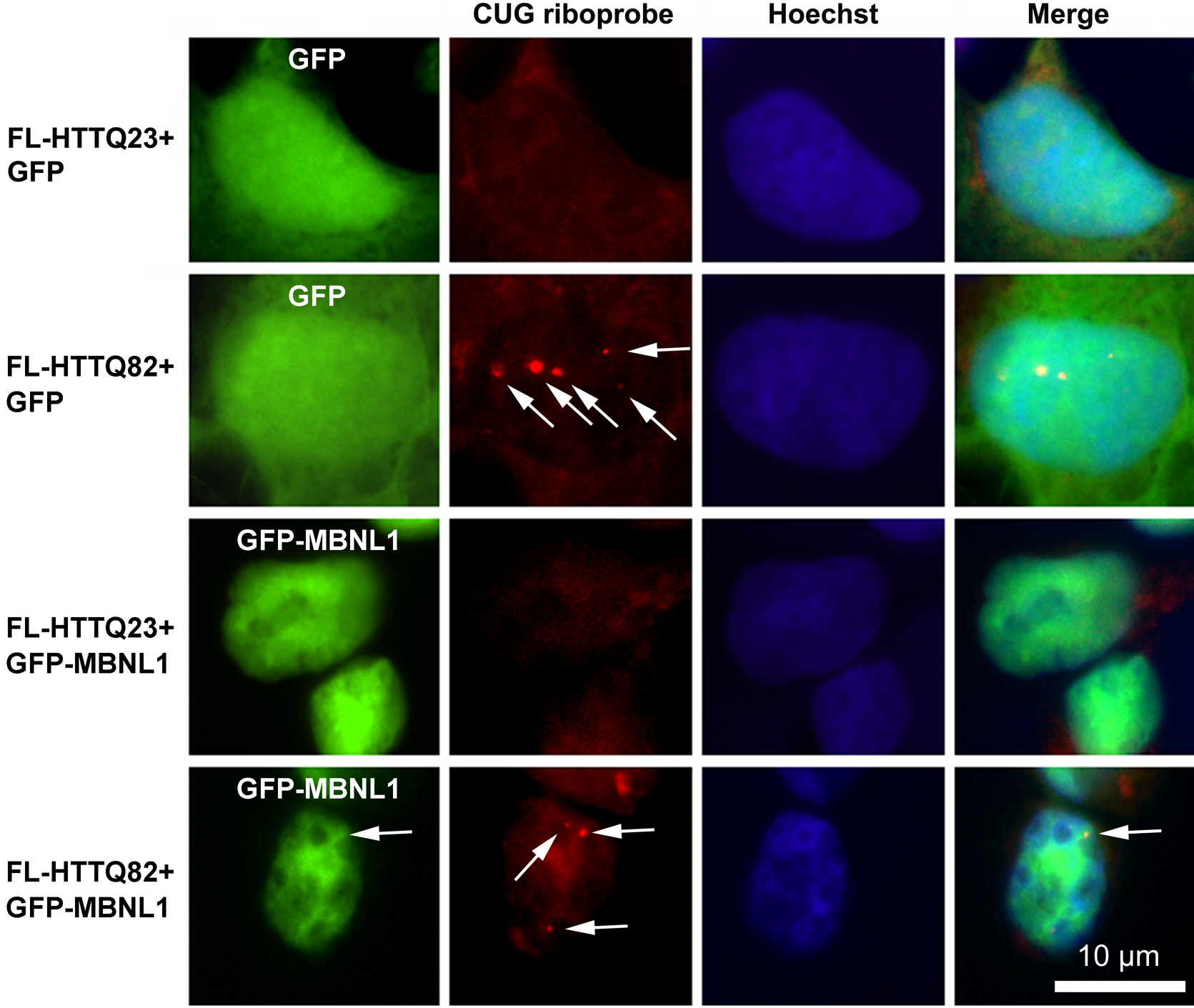
**

**Fig. S3 Representative images used in RNA foci analysis.** SK-N-MC cells were co-transfected with FL-HTT and GFP-MBNL1 plasmids and subjected to FISH 48 hours post transfection. GFP plasmid was used as a control. Scale bar, 10 μm. Green fluorescence showed expression of GFP or GFP-MBNL1 in cells. Foci were visualized by red fluorescence (arrows). Hoechst was used as a nuclear marker. Note that GFP-MBNL1 co-localized with *FL-HTTQ82* RNA foci (bottom row). Scale bar, 10 μm.

**
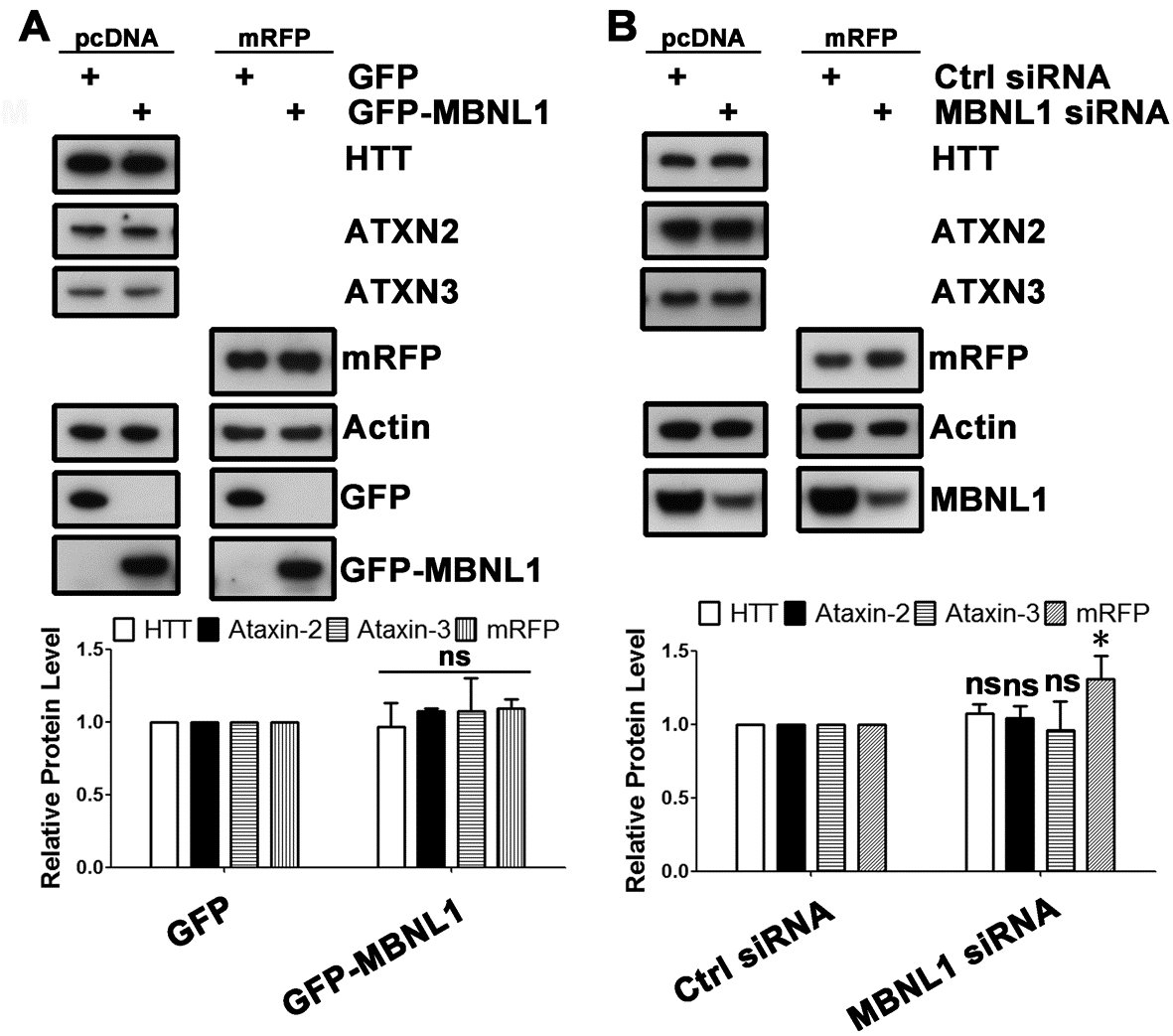
**

**Fig. S4 Overexpression of MBNL1 has no effect on endogenous control proteins with normal CAG repeats or exogenous proteins without CAG repeats.** (**A**) SK-N-MC cells were co-transfected with mRFP and GFP-MBNL1 plasmids, and the levels of proteins were assessed by western blot 72 hours post-transfection. pcDNA3.1 plasmid was used as a control. Overexpression of MBNL1 had no effect on levels of endogenous HTT, ATXN2 or ATXN3 encoded by normal CAG repeats. The level of exogenous mRFP (no CAG repeats) was not changed by overexpression of MBNL1. Student’s *t*-test, n=3 biological replicates. ns=no significance, versus GFP group. (**B**) SK-N-MC cells were first transfected with MBNL1 siRNA and 72 hours later cell were further transfected with mRFP plasmid and incubated for additional 48 hours. Levels of the above proteins were assessed by western blot. Control siRNA was used as a control. Knock-down of MBNL1 did not change the levels of endogenous HTT, ATXN2 or ATXN3, but mildly increased the level of exogenous mRFP. Student’s *t*-test, n=3 biological replicates. *P<0.05, ns=no significance, versus control siRNA group.

**
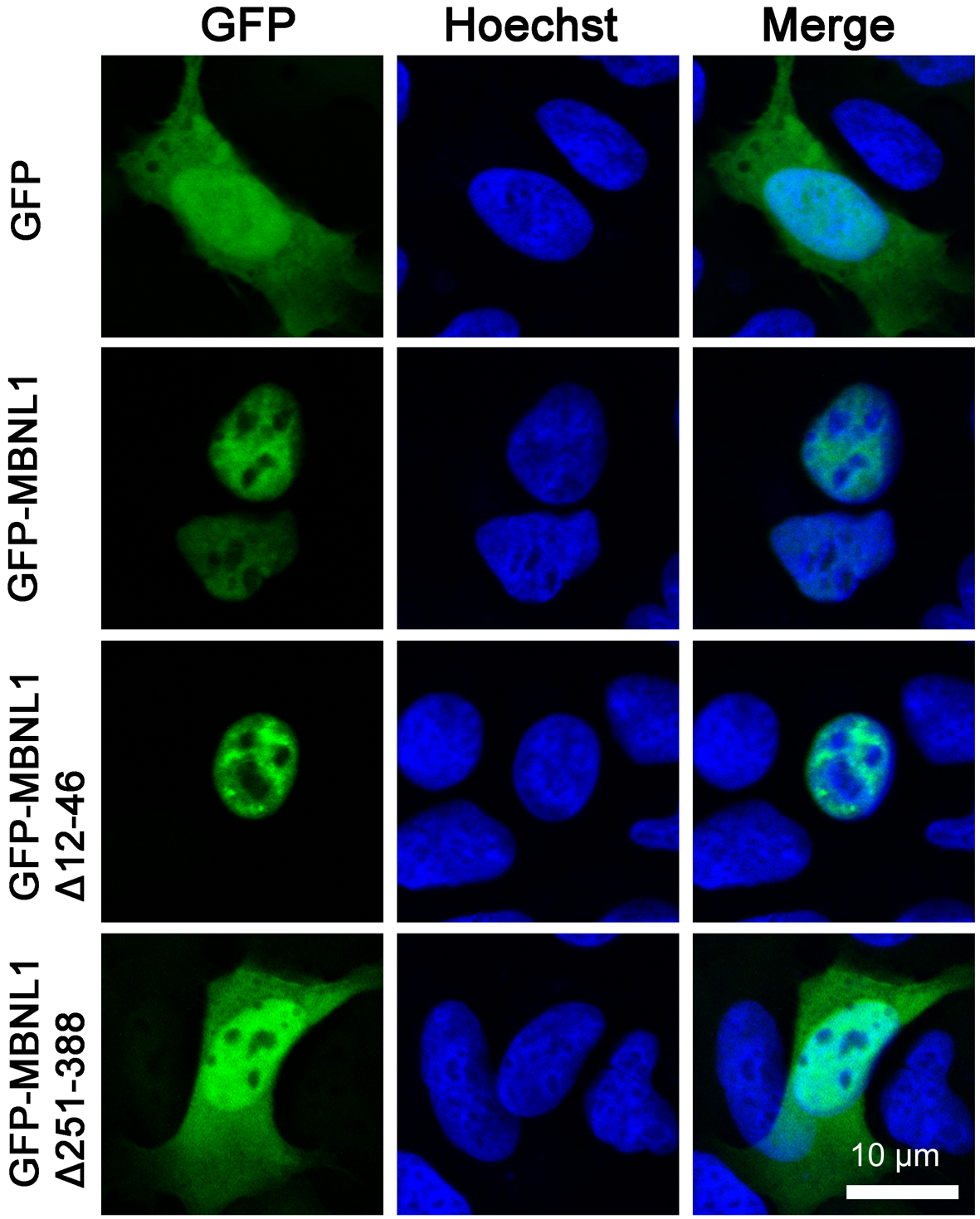
**

**Fig. S5 Cellular localization of GFP-MBNL1.** SK-N-MC cells were transfected with GFP-MBNL1 plasmids, and cellular localization of GFP-MBNL1 variants was imaged by confocal microscopy 24 hours post-transfection. Hoechst was used as a nuclear marker. GFP-MBNL1 and GFP-MBNL1 Δ12-46 were mainly expressed in the nucleus, whereas GFP-MBNL1 Δ251-388 was distributed in both the cytoplasm and nucleus. Scale bar, 10 μm.

**
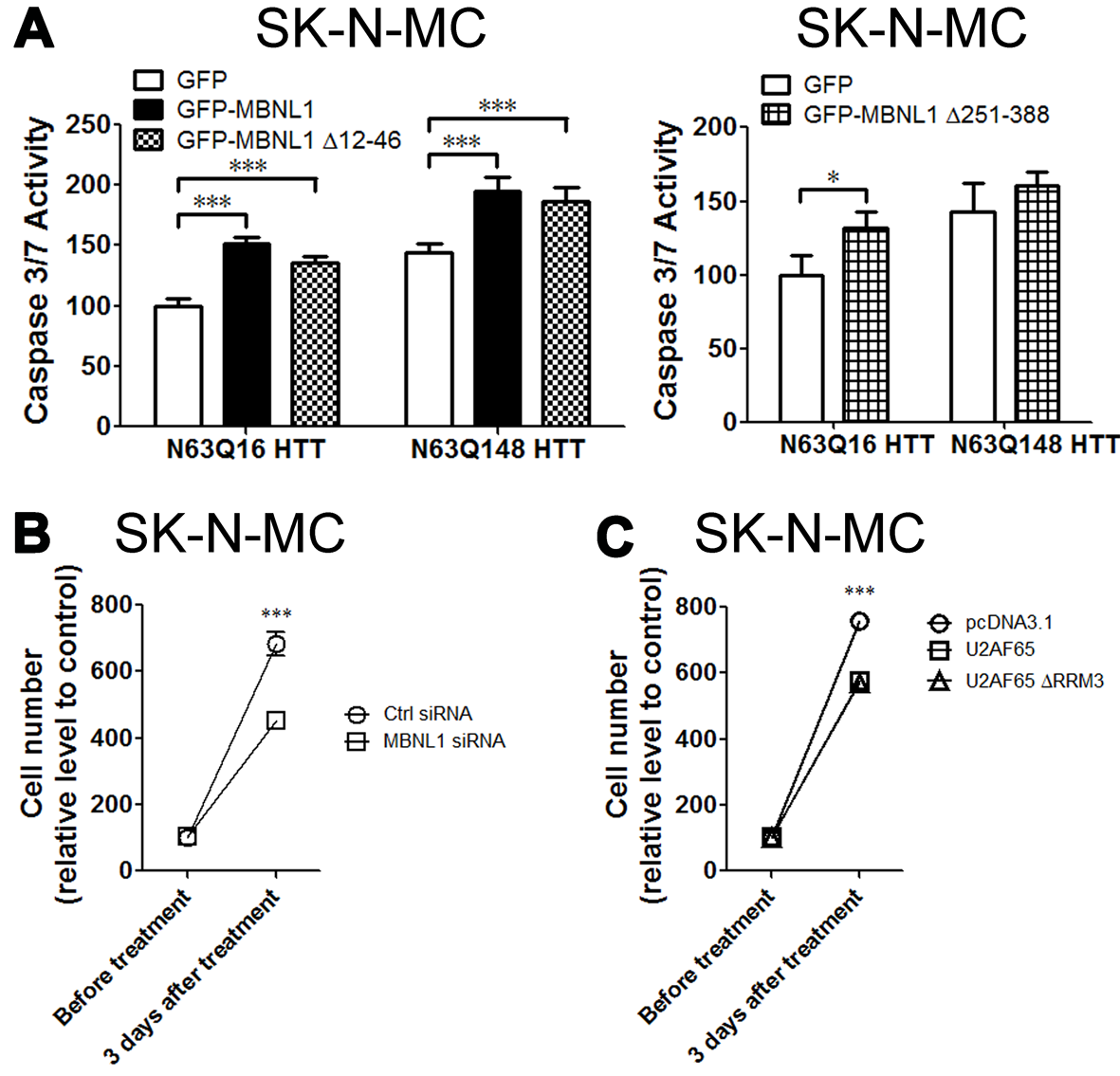
**

**Fig. S6 MBNL1 and U2AF65 homeostasis is critical for cell viability.** (**A**) MBNL1, MBNL1 Δ12-46 and MBNL1Δ251-388 are toxic to normal and HTT-expressing SK-N-MC cells. Both experiments, two-way ANOVA, n=4 biological replicates. *P<0.05, ***P<0.001. (**B**) Knock-down of MBNL1 is cytotoxic. SK-N-MC cells were transfected with human MBNL1 siRNA, and the cell numbers before and after transfection were examined by MTT proliferation assay 72 hours post-transfection. Control siRNA was used as a negative control. Two-way ANOVA, n=4 biological replicates. ***P<0.001, MBNL1 siRNA group versus control siRNA group. (**C**) Overexpression of both U2AF65 and U2AF65 ΔRRM3, lacking the most C-terminal RNA recognition motif (RRM) domain, triggers cytotoxicity in normal SK-N-MC cells. SK-N-MC cells were transfected with U2AF65 or U2AF65 ΔRRM3 plasmid and the cell numbers before and after transfection were examined by MTT proliferation assay 72 hours post-transfection. pcDNA3.1 plasmid was used as a negative control. Two-way ANOVA, n=4 biological replicates. ***P<0.001, U2AF65 group or U2AF65 ΔRRM3 group versus pcDNA3.1 group. Both knock-down of MBNL1 and overexpression of U2AF65 inhibited proliferation of SK-N-MC cells.
